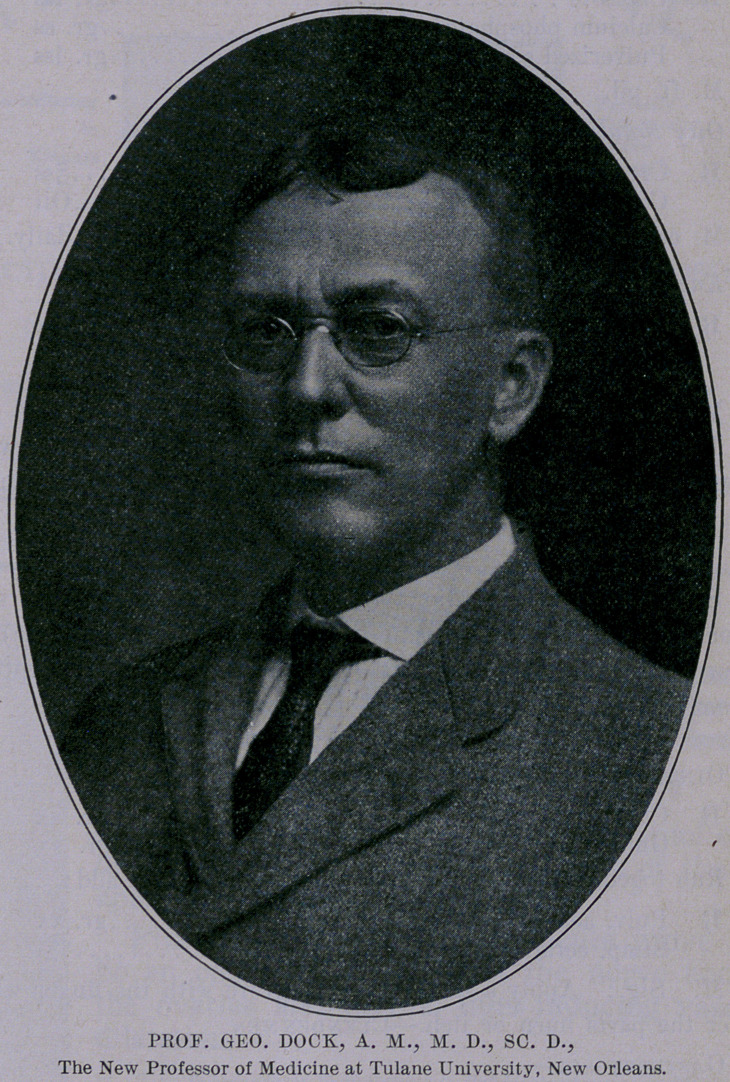# The Regulation of the Social Evil in Our Large American Cities

**Published:** 1908-11

**Authors:** G. Frank Lydston

**Affiliations:** Chicago, Professor of Genito-Urinary Surgery, University of Illinois


### TEXAS MEDICAL JOURNAL.

Established July, 1885.

F. E. DANIEL, M. D., - - - - Editor, Publisher and Proprietor

PUBLISHED MONTHLY.—SUBSCRIPTION $1.00 A YEAR.

VOL. XXIV. AUSTIN, NOVEMBER, 1908. No. 5.

The publisher is not responsible for the views of contributors.

### Original Articles.

For Texas Medical Journal.

### The Regulation of the Social Evil in Our Large American Cities.

BY G. FRANK LYDSTON, M. D., CHICAGO,
Professor of Genito-Urinary Surgery, University of Illinois.

In a recent issue of a Chicago paper, in connection with a discussion of graft in our municipal governmental system, it was stated that the "Graft Commission" was considering seriously the advisability of legal regulation and sanitary control of the social evil. Much argument was advanced in behalf of segregation.

The sudden alarming discovery by the innocents of the Graft Commission that such a thing as the social evil exists in Chicago would be amusing were it not for the seriousness with which they take themselves as guardians of the public weal. The desire of the Commission to protect the police from the temptation to blackmail by regulating, inspecting and controlling the victims of police blackmail would strike the unprejudiced observer as a reflection on the intelligence of the Commission. The members of the Graft Commission, and incidentally the newspapers of Chicago, seem to have awakened very suddenly to the existence of evils of which the average intelligent citizen has been perfectly cognizant time out of mind. That the newspaper fraternity has been ignorant of the real conditions is doubtful and a reflection on its discernment.

It would be superfluous at this time to attempt an exhaustive discussion of the social evil and the remedies for it. I shall, however, probably do so later—but, as a student of sociology, I feel that it would not be out of place for me to make certain comments upon the suggestion of restriction and regulation. I use Chicago merely as a near-to-hand illustration. My remarks apply to American cities in general.

Has regulation, or segregation, or medical inspection thus far proven brilliantly successful in the countries in which it has been established? Statistics have been juggled with by those on both sides of the argument, and each side has, apparently, in some instances at least, proven its point. The most definite results thus far obtained have been in military garrisons. Chicago, I might remark, is not a military garrison, nor is it a small town, nor is its population under military control and surveillance. When such a man as Fournier, of Paris, states his belief as essentially being that statistics are valueless upon the point in question, serious reflection would seem to be indicated. Unbiased, unprejudiced investigators—such, for instance, as the New York Committee of Fifteen—have summed up the results of their investigations in these words: "Regulation does not regulate; segregation does not segregate."

Is it possible to submit prostitutes to regulation, restriction and inspection? It is variously claimed that the proportion of unregistered women, or "clandestines," in the large cities of Europe is ten or fifteen times that of the registered. In 1890, Niemann estimated the number of prostitutes in Berlin at fifty thousand. The statistics taken two or three years before this period show less than four thousand under sanitary control. Competent observers have stated that clandestines are much more dangerous to society than "professionals." Regulation has been proven to lessen the proportion of professionals and increase the proportion of clandestines.

Even admitting, for the sake of argument, the success of regulation in Europe, conditions in America are different from those abroad. Personal liberty is much more jealously guarded here, and paternalism is looked upon with less friendliness. To register your individual, you must first do as an old college professor said must be done in making chicken soup: "First, catch your chicken." Apropos of results in Europe, it might be remarked that after a hundred years of police regulation in Paris, the authorities practically confess their inability to cope with the social evil.

Regulation, inspection and control will be very expensive. Who will "pay the freight"—the women themselves? They would probably be as much averse to a systematic legal tax as they are to the system of blackmail that is at present inflicted upon them. The least that could result would be the scattering of the poor creatures into various portions of the city, where they could ply their trade clandestinely.

Shall the reputable citizen pay the tax? The reputable citizen,

DR. G. FRANK LYDSTON, CHICAGO.

dizzily teetering upon his pinnacle of self-labeled propriety, would probably object to paying the tax for the benefits of individuals who, theoretically, at least, he considers not quite so proper as himself. Least of all would the numerous whited sepulchers of our social system want to pay the tax.

So far as "medical inspection" is concerned, to the medical profession it is simply nauseous. Shall the medical profession constitute itself an assurance association, the function of which is to guarantee, in a measure, immunity from the penalties of vice? Medical science is by no means infallible. It is practically impossible to carry out medical inspection with sufficient thoroughness to be of much practical value; and the implied guarantee of safety would increase the patronage of vicious resorts 100 per cent. I presume there would be no difficulty in finding physicians who would be willing to stoop to the plane of inspector of bagnios. High professional ideals and hungry stomachs are often incompatible. Besides, brains, and particularly medical brains, are about the cheapest stuff on the market. It is bad enough for the medical profession to be placed in the position in which it is at the present time and be the sole guardian and savior of an ungrateful public from the results of vice, without asking the profession to unite the rôle of healer to that of a guarantee company, the function of which shall be to assure the safety of vicious indulgence. If there is a lower or more degrading function which a medical man can be asked to perform, than that involved in medical inspection of the denizens of the tenderloin, I do not know what it is. I presume that in these halcyon days of combination the union of the city of Chicago, the keeper of bagnios and the medical man who is willing to offer the guarantee elements of a social evil trust would be very formidable. The natural outgrowth of it would be, however, a new trades union, which would involve the people regulated. Any official recognition, by means of licensing of or tax gathering from prostitutes, gives the official sanction of society to the social evil and puts it on a plane of legitimacy with other occupations. It is well enough to recognize the social evil as unavoidable; to recognize it as necessary is decidedly open to criticism.

Segregation would simply result in the establishment of a certain quarter of the city which would be a moral plague spot, which sooner or later would embrace the thieves and cut-throats as well as the tenderloin population proper. Experience has shown that it is impossible to confine the social evil within certain definite limits. The attempt to do so would restrict the facility with which the patrons of such districts are wont to go and come, as a consequence of which the social evil would simply be driven into more respectable localities. Again, a segregation law would be a formidable "mace" for the brass-button blackmailer.

The most that is practicable in dealing with the social evil is to prevent prostitutes from flaunting themselves before the public gaze. Any custom that makes their trade conspicuous is deleterious to the welfare of society, and should be promptly suppressed. The nymphs of the pave should be given their choice between the seclusion of the bagnio and honest labor. There must, however, be some provision or opportunity for honest labor. If such poor creatures are driven off the streets, there are but two possible avenues of escape open to them: the bagnio, which is already full to overflowing, and the river. They must live; and to drive them off the streets permanently is likely to result in starvation. To demand that they live respectable lives without giving them any opportunity to do so is about as logical as the advice I once heard a young hospital interne give a consumptive patient in the Blackwell's Island Penitentiary Hospital. He said: "My friend, what you need is a trip to Colorado."

If the Graft Commission is really in earnest and would like to know the facts with regard to the police and the social evil, let them investigate the occasional round-ups of the inmates of the bagnios in the levee district. The "straw bailer," the captain of the precinct, the police justice and the patrolmen know the conditions in the tenderloin district so thoroughly that the periodic round-ups of these women have been most beautifully adapted to their relative degree of fornicatorial prosperity. I have seen the cells at the Harrison Street station full of these women who had been captured on a certain evening and who, so far as could be learned, had committed no offenses on that particular evening in anywise different from those which they had committed for many evenings before. With extreme generosity, the powers that be gave the women time enough to earn sufficient money to pay their fines and the "commission" fee of the straw bailer. Many who were not caught in the net enjoyed an immunity which proves that the police department could explain some queer things if investigated thoroughly.

---

### Dr. George Dock.

Dr. George Dock graduated from the medical department, University of Pennsylvania, in 1884; interne in St. Mary's Hospital, Philadelphia, for a year, under Drs. W. W. Keen, John B. Roberts, J. Ewing Mears and others; studied in Leipsic, Berlin, Vienna and Frankfort from 1885 to 1887. From 1887 to 1888 he was assistant in charge of the laboratory of clinical medicine in the University of Pennsylvania, under Drs. William Osler and John H. Musser. At the same time he was also physician to the medical dispensaries of the University Hospital and St. Agnes' Hospital, in the former working in connection with Dr. William Pepper. In Philadelphia Dr. Dock began investigations in malaria, with Osler, who was one of the earliest to follow the discoveries of Laveran, and he also studied typhoid fever. In 1888 he became Professor of Pathology and Clinical Medicine in the Galveston Medical School, where he made studies in malaria, dysentery, typhoid fever and other diseases, often referred to in articles and textbooks on those subjects. In 1891 he was appointed Professor of Medicine in the University of Michigan, and made important advances in the methods of teaching in that celebrated school, such as instituting practical courses in various departments of internal medicine, and doing away with repetition of lectures and establishing the promotion of the reading and recitation method, the diagnostic and the therapeutic clinic, and expanding the work in both wards and hospital laboratories. The effects of the change are attested by the practical and at the same time scientific work of graduates of the school generally. Dr. Dock has always been an investigator, making many contributions to all divisions of medicine, especially in diseases of the blood, goitre, exophthalmic goitre and other diseases of the ductless glands, protozoan diseases, etc. He has contributed many articles to medical periodicals and encyclopedias, and edited the volume on the Heart in the American issue of Nothnagel's System. He is considered an authority on vaccination, and was selected to write the chapter on that subject in Osler's Modern Medicine. In 1898 he spent some time in army camps at Chickamauga, Knoxville, and elsewhere, investigating camp fevers for the government. Dr. Dock is a member of many medical societies and of the American Association for the Advancement of Sciences, and of the scientific society of Sigma Xi. Was delegate to the Congress *für innere Medezin,* held in Berlin, and the International Medical Congress at Moscow, in 1897; at the latter was one of the vice-presidents of the Section on Medicine. In 1901 was honorary vice-president of the International Congress on Tuberculosis, in London, and is a member of the International Congress on Tuberculosis just held in Washington in September, being also a member of the Committee on Prizes and also of the Hodgkins Prize of the Smithsonian Institute.

He has held various positions in the American Medical Association, as secretary and chairman of the Section on Medicine, and as orator in medicine; is chairman of the Editorial Board of the

*Archives of Internal Medicine.* Dr. Dock received the honorary degree of A. M. at Harvard University in 1895, and the Sc. D. at Pennsylvania in 1904, as a recognition of his distinguished work in medicine; he gave the address on Pathology at the dedication of the great laboratories of the medical college of the University of Pennsylvania.

PROF. GEO. DOCK, A. M., M. D., SC. D.,

The New Professor of Medicine at Tulane University, New Orleans.

Dr. Dock comes to New Orleans as no stranger, for his stay in Galveston made him many friends in and out of the profession, and the fact that his accomplished wife is a Southern woman, native of Galveston, should give both a warm welcome to New Orleans.—*N. O. Med. and Surg. Journal,* October, 1908.

Methods of Using Creosote.—Creosote, alone or combined with calcium phosphate, sodium arsenate, etc., may be given either in pill form or in oily solutions and in enemata, as in the following forms:

*Pills:*

℞ Creosote . . . . . . . . . . . . . . . . . . . . . . . . . . . . . . . . gr. iss
Calcium phosphate . . . . . . . . . . . . . . . . . . . . . . . . gr. ss
Pulverized soap . . . . . . . . . . . . . . . . . . . . . . . . . gr. iss

M. ft. pil.

*Oily Solution:*

℞ Creosote . . . . . . . . . . . . . . . . . . . . . . . . . . . . . . . . . ℥ss
Cod liver oil . . . . . . . . . . . . . . . . . . . . . . . . . . . . . . Oii

M. et sig.: One tablespoonful twice or three times daily.

*Enema:*

℞ Olive oil . . . . . . . . . . . . . . . . . . . . . . . . . . . . . . . . ℥x

Creosote . . . . . . . . . . . . . . . . . . . . . . . . . . . . . . . . . ℥i

Wine of opium . . . . . . . . . . . . . . . . . . . . . . . . . . . Mxlv

M.

Two tablespoonfuls of this mixture added to a glassful of warm water are emulsified with the yolk of an egg to form an enema.—*La Clinique,* July 3, 1908.

---

Strong antiseptic solutions should be avoided in dressing scalp wounds. For "wet dressings" Thiersch's (boro-salicylic) or Burow's (aluminum acetat) solution is sufficiently antiseptic.—*American Journal of Surgery.*

---

Ointment for Pruritus Vulvae.—

℞ Chloral hydrate . . . . . . . . . . . . . . . . . . . . . . . . . . gr. xx
Gum camphor . . . . . . . . . . . . . . . . . . . . . . . . . . . gr. xx

Rub together in a mortar until they liquefy, then add:

℞ Pulv. acaciæ . . . . . . . . . . . . . . . . . . . . . . . . . . . gr. xx
Simp. cerat . . . . . . . . . . . . . . . . . . . . . . . . . . . . . . ℥i

M. Sig.: Apply a little of the mixture with the finger whenever the parts burn or itch.—Clendennen.

Gastroptosis.—

℞ Chloral hydrat . . . . . . . . . . . . . . . . . . . . . . . . . . . . . ʒi

Sodii bromidi . . . . . . . . . . . . . . . . . . . . . . . . . . . ʒiiss

Aq. chloroformi . . . . . . . . . . . . . . . . . . . . . . . . . . ℥iv

Spts. anisi . . . . . . . . . . . . . . . . . . . . . . . . . . . . gtt. vi

M. Sig.: Teaspoonful in water after meals three times a day. —Lockwood.

## Figures and Tables

**Figure f1:**
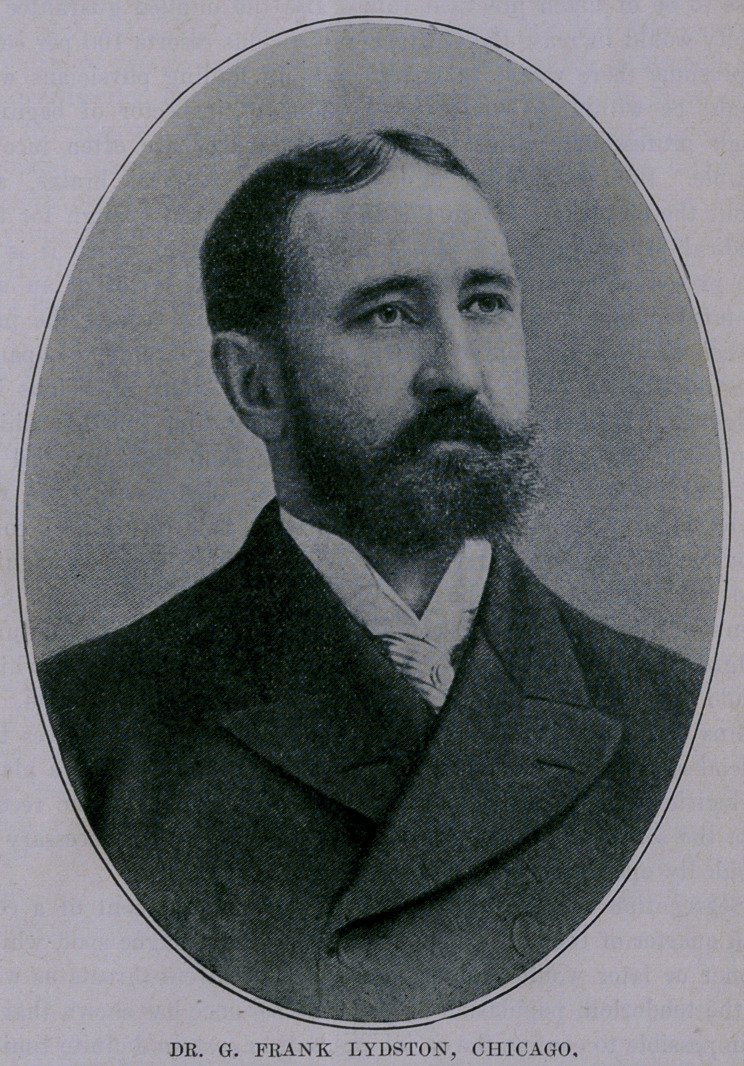


**Figure f2:**